# Normal Fibroblasts Induce E-Cadherin Loss and Increase Lymph Node Metastasis in Gastric Cancer

**DOI:** 10.1371/journal.pone.0097306

**Published:** 2014-05-20

**Authors:** Wen Xu, Xinlei Hu, Zhongting Chen, Xiaoping Zheng, Chenjing Zhang, Gang Wang, Yu Chen, Xinglu Zhou, Xiaoxiao Tang, Laisheng Luo, Xiang Xu, Wensheng Pan

**Affiliations:** 1 Department of Gastroenterology, Second Affiliated Hospital of Zhejiang University, School of Medicine, Hangzhou, China; 2 Department of Orthopedics, Second Affiliated Hospital (Binjiang Branch) of Zhejiang University, School of Medicine, Hangzhou, China; 3 Department of Pharmacy, Second Affiliated Hospital of Zhejiang University, School of Medicine, Hangzhou, China; 4 Department of Pathology, Qujiang People’s Hospital, Quzhou, China; 5 Zhejiang Academy of Traditional Chinese Medicine, Experimental Animal Research Center, Hangzhou, China; 6 Department of Pharmacy, Second Affiliated Hospital (Binjiang Branch) of Zhejiang University, School of Medicine, Hangzhou, China; 7 Department of Gastroenterology, Second Affiliated Hospital (Binjiang Branch) of Zhejiang University, School of Medicine, Hangzhou, China; AMS Biotechnology, United Kingdom

## Abstract

**Background:**

A tumor is considered a heterogeneous complex in a three-dimensional environment that is flush with pathophysiological and biomechanical signals. Cell-stroma interactions guide the development and generation of tumors. Here, we evaluate the contributions of normal fibroblasts to gastric cancer.

**Methodology/Principal Findings:**

By coculturing normal fibroblasts in monolayers of BGC-823 gastric cancer cells, tumor cells sporadically developed short, spindle-like morphological characteristics and demonstrated enhanced proliferation and invasive potential. Furthermore, the transformed tumor cells demonstrated decreased tumor formation and increased lymphomatic and intestinal metastatic potential. Non-transformed BGC-823 cells, in contrast, demonstrated primary tumor formation and delayed intestinal and lymph node invasion. We also observed E-cadherin loss and the upregulation of vimentin expression in the transformed tumor cells, which suggested that the increase in metastasis was induced by epithelial-to-mesenchymal transition.

**Conclusion:**

Collectively, our data indicated that normal fibroblasts sufficiently induce epithelial-to-mesenchymal transition in cancer cells, thereby leading to metastasis.

## Introduction

Metastases are responsible for up to 90% of cancer-associated mortality. Many patients who demonstrate no evidence of metastasis at the initial diagnosis will eventually develop metastasis. Although metastases cause most cancer deaths, this process remains one of the most enigmatic aspects of the disease.

Metastatic tumor cells enter tissue via extravasation. The tissue, however, undergoes unclear processes by which the cells are imbedded in the tissue matrix and come into direct contact with stromal cells, most of which are normal fibroblasts. Tumor cells have every chance to come into contact with stromal cells, including neoplastic and metastatic cells [1]. This leads to reciprocal cross-talk with normal fibroblasts in the tissue.

Fibroblasts are the most abundant stromal cells, and they stimulate the microenvironment and serve as a rich source for the paracrine pathway during tumorigenesis and progression. The effects of normal fibroblasts on tumor cells are disputed. It has been reported that normal fibroblasts suppress the malignant conversion of immortalized prostate epithelium [2], whereas in breast tumors, the fibroblasts transform ductal carcinoma into invasive carcinoma [3].

This disagreement indicates that the influence of fibroblasts on tumor cells is different than for CAFs (cancer associated fibroblasts). For this study, we cocultured tumor cells with normal fibroblasts in Petri dishes in order to mimic how tumor cells contact fibroblasts. We focused on the contribution of normal fibroblasts to gastric cancer. We cultured the normal fibroblasts to form dense monolayers, which were then disseminated with tumor cells in order to mimic metastatic tumor cells. We hypothesized that the high ratio of fibroblasts to tumor cells might mimic the tissues where metastasized tumor cells reside. Thus, dermal fibroblasts from healthy individuals were used to produce the cellular environment. The gastric cancer cell line, BGC-823, was used here because it is morphologically distinct from fibroblasts.

## Materials and Methods

### Ethical Statement

Primary human dermal tissues were obtained from children who underwent circumcision after obtaining written informed consent from their caretakers, which was included in their medical records. This experiment was reviewed and approved by the institutional review board of Second Affiliated Hospital of Zhejiang University School of Medicine (ethical review code: Research 2013-047). The patients included in this study were provided with a copy of written informed consent and gave permission to publish.

All experiments were conducted according to the Guidelines for the Care and Use of Laboratory Animals of the Council of Science and Technology of China. The study protocol was approved by the Animal Care and Use Committee of Zhejiang University.

### Reagents and Antibodies

Penicillin G/streptomycin, phosphate-buffered saline (PBS), Triton X-100, bovine serum albumin, collagenase type I, and trypsin were purchased from JiNuo (China). High-glucose DMEM was obtained from Gibco (China), and fetal bovine serum (FBS) was purchased from Gibco (SA). Cisplatin, 5-fluorouracil, puromycin, and collagen type I were purchased from Sigma (USA). Cisplatin was dissolved in dimethylformamide (DMF), 5-fluorouracil in DMSO, and puromycin in PBS in order to make stock solutions that were then stored at −20°C. Collagen type I (5 mg/mL) was diluted to 1 mg/mL. DAPI (4,6-diamidino-2-phenylindole·HCl) in PBS was obtained from Roche, and goat anti-mouse and rabbit IgG, secondary antibody-TIRTC, and secondary antibodies were obtained from ZSGB-Bio (China). Antihuman-E-cadherin rabbit (ab40772), antihuman-vimentin rabbit (ab92547), and antihuman-β-catenin rabbit antibodies (ab9274) were obtained from Abcam (UK). Antihuman-pan-CK mouse antibody (C11) and N-cadherin rabbit antibody (C4061) were obtained from Cell Signaling Technology (China), and anti human-β-actin and secondary goat anti-rabbit antibodies were obtained from Boster (China). Antibodies were diluted with 5% FBS to working concentrations unless otherwise mentioned.

### Cell Culture

The established cell line, human gastric cancer BGC-823 cells used in our experiment were purchased from the cell bank of Guangzhou Institute Biomedicine and Health. The cells were thawed and passaged for 4–5 times and then were used in the co-culture experiments. Primary dermal fibroblasts were obtained from the surgical specimens of children who had undergone circumcision and provided informed consent. Fibroblasts were used after the third passage (within 50 passages), cultured in high-glucose DMEM containing 10% FBS, and maintained in a humidified incubator (5% CO_2_) at 37°C. The cell medium was replaced every other day.

For the generation of the TBGCs, the fibroblasts (FBs) and BGC-823 cells were cocultured at a ratio of 10∶1 for an additional 10 days after the cultured cells reached confluence. Dome formation was observed in the co-culture system. The cell mixture was then passaged by trypsinization with fresh fibroblasts (1×10^6^ cells/mL). During the second and subsequent passages, apparent doom formation and the suspended round-shaped cells appeared which exhibited diverse morphological features and prolonged attachment after passage. After the fifth passage of the mixed suspended cells and dense normal fibroblasts, uniform, short, spindle-like cells termed “TBGCs” were produced and did not demonstrate morphological reversion to BGC-823 cells until >10–15 passages.

### Proliferation Assay

Exponentially growing cells were seeded in 96-well plates for an additional 6 days of incubation at 37°C in a humidified 5% CO_2_ atmosphere. Cell proliferation was measured in quintuplicate every day by pipetting 20 µL CellTiter 96 AQueous One Solution Reagent (Promega, USA) into each well that contained 100 µL high-glucose DMEM, then cells were incubated for another 2 hours before determining the relative absorbance at 490 nm using a microtiter plate reader.

### Drug Inhibition Assay

Five duplications of exponentially growing cells were seeded in a 96-well plates and incubated for 24 hours. After 24 hours, the medium was replaced with different concentrations of drugs (0–80 µM) and cultured for an additional 24 hours. Drug toxicity was evaluated by measuring the number of viable cells using the proliferation assay described above.

### Scratching Assay

Cells were seeded onto 24-well plates (5×10^4^ cells/well) and cultured to confluence. Pipette tips were used to generate scratches. PBS was used to remove cell debris, then replaced with FBS-free medium. Images were obtained for 3 consecutive days. The width of the scratch was measured using ImageJ 1.47 software for Windows (http://rsb.info.nih.gov/ij/) and plotted as the scratch distance per day.

### Migration and Invasion Assay

Cell motility assaying was performed using transwell inserts (8 µm pore size) (Corning, USA). Cells-GFP (Green Fluorescent Protein) (1×10^5^/500 µL) were suspended in FBS-free medium and placed above the inserts in triplicate, with 800 µL culture medium loaded underneath. After overnight incubation, the inserts were washed 3× with PBS and wiped with a wetted swab above where fixed with 4% PFA below and observed using a fluorescent microscope (Olympus, Japan). The same procedure was used to perform the invasion assay, but inserts that were precoated with Matrigel (BD, USA) were used. Both assays were quantified as the number of cells counted in 5 random fields (200×magnification).

### Apoptosis Assay

Apoptotic cells were measured using the Annexin V-FITC apoptosis detection kit (Keygen, China) according to the manufacturer’s instructions. Cells were either untreated or treated with drugs for 24 hours, and then harvested, washed twice with PBS, resuspended in binding buffer containing 5 µL FITC-Annexin V and 5 µL PE, and incubated at room temperature for 30 minutes. Analysis was performed using flow cytometry (FACSCalibur, Becton Dickinson, USA).

### Quantitative Real-time PCR

Nuclear extracts were prepared using the RNeasy mini-kit according to the manufacturer’s instructions. Purified RNA samples (1 µg) in DEPC water were reverse-transcribed using the PrimeScript II 1st Strand cDNA Synthesis Kit (Takara, China). RT-PCR and amplification were performed using the SYBR Premix Ex Taq II kit (Takara).

A 20 µL reaction system containing 1 µL diluted cDNA samples, 10 µL SYBR Premix Ex Taq II (2×), 0.4 µL ROX Reference Dye (50×), 7 µL sterile double distilled H_2_O, and 1 µL forward and reverse primers (see Materials and Methods S1) were prepared and evaluated using melting curve analysis and the comparative threshold cycle (Ct) method. The following cycling conditions were used: 95°C for 10 minutes, followed by 40 cycles of 95°C for 15 seconds and 58°C for 1 minute. GAPDH was used as the control gene.

### Western Blot Analysis

Proteins were extracted from exponentially growing cells and primary tissues. The extracts were boiled in loading buffer, resolved on 10% Tris-HCl SDS-polyacrylamide gels, and transferred to nitrocellulose membranes (Millipore, USA) using standard methods. The membranes were blocked using 5% fat-free milk in Tris-buffered saline with tween-20 (TBST) for 30 minutes at room temperature. Membranes were washed with TBST and incubated overnight at 4°C with primary antibodies against E-cadherin (1∶5000), vimentin (1∶5000), β-catenin (1∶5000), N-cadherin (1∶1000), and β-actin (1∶1000). After washing 3× with TBST, secondary goat anti-rabbit (1∶1000) was added and incubated for 2 hours at room temperature. Finally, immune complexes were visualized by chemiluminescence using ECL (Electro Chemical Luminescence) (Millipore). The protein concentration was normalized to β-actin.

### Histological and Immunohistochemical Analyses

Samples were quickly frozen in liquid nitrogen, stored at −80°C, fixed in 4% PFA, and sectioned to a thickness of 10 µm (LEICA, Germany).

For hematoxylin and eosin (HE) staining, the deparaffinized slides were stained with hematoxylin for 15 minutes, washed 3× with PBS, acid alcohol for 5 seconds, then eosin for 5 minutes (Boster, China).

For immunohistochemical staining, the slides were rehydrated and heat-induced epitope retrieval was performed in citrate buffer using a pressure cooker (20 minutes at 80 pKA), blocked with 3% H_2_O_2_ for 15 minutes, and then normal goat serum was applied (30 minutes at room temperature). Slides were incubated in the following primary antibodies: anti-E-cadherin (1∶250), anti-vimentin (1∶250), anti-β-catenin (1∶250), and anti-pan-CK (1∶250). Samples were incubated overnight at 4°C, rinsed twice with PBS, and incubated with the secondary antibody for 2 hours at 37°C. DAB Plus Chromogen kit (Boster) was used to detect all antigens. The slides were counterstained with hematoxylin, dehydrated, and mounted with coverslips using neutral balata. Both analyses were separately performed by pathologists and clinicians who were blind to the sample groups. Disputes were resolved by consensus.

### Immunofluorescence and Confocal Microscopy

Cells were plated onto chamber slides, and the frozen sections were fixed with 4% PFA and permeabilized with 0.5% Triton X-100. Slides were blocked with normal goat serum for 1 hour at 37°C, rinsed and incubated overnight with primary antibodies at 4°C, then transferred and incubated with goat anti-mouse and anti-rabbit-TIRTC antibodies for 1 hour at 37°C in the dark. Slides were washed using glycerin and mounted with cover slips. Nuclei were counterstained with DAPI. Fluorescence was detected using a confocal laser-scanning microscope (Olympus). The deparaffinized cryosections were stained with DAPI and analyzed using an immunofluorescent microscope.

### Animal Model

Eighty four-week-old female BALB/c mice were purchased from the Animal Research Center of Shanghai and maintained at the experimental animal center of the Zhejiang Academy of Medical Science. According to the Guidelines for the Care and Use of Laboratory Animals of the Council of Science and Technology of China, all animals were kept under aseptic sterile conditions and administered autoclaved food and water in compliance with the Principles of Laboratory Animal Care of Zhejiang University. Forty-four mice were used in the subcutaneous model, and were divided evenly into control and experiment groups. The BGC-GFPs were used as control where the TBGC-GFPs were uses as the experiment. The tumor cells were harvested during the growth phase, and suspended in FBS-free medium (1×10^6^ cells/mL). Cells were pipetted to single-cell suspensions, and each 500 µL solution was subcutaneously inoculated at both sides of the chest. Mice were euthanized every 2 weeks till 10 weeks, and pathological examinations were performed. All the mice were weighed every 3 days. All surgeries were performed under 1% sodium pentobarbital, and all efforts were made to minimize suffering.

Thirty-six mice were used in vein injection group for the assessment of cancer distribution (18 mice in BGC-GFP control group and 18 mice in TBGC-GFP experiment group). 500 µL cell suspension (5×10^5^ cells) was injected into the tail vein of nude mice. Mice were euthanized at 2^nd^, 5^th^, 8^th^ and 10^th^ weeks for pathological examination.

### Statistical Analysis

Experimental data were collected and entered into spreadsheets. Normality tests were performed before comparing the data. Comparisons between groups were performed using Student’s *t* test. One-way analysis of variance was used to compare>3 groups, and Tukey’s method was used for *post hoc* comparisons of groups. In this study, *p*<0.05 is considered statistically significant. SPSS 20.0 for Windows (IBM SPSS Statistics, http://www-01.ibm.com/support/docview.wss?uid=swg24029274) was used to perform all statistical analyses. R (ggplot2) (http://www.r-project.org/; http://www.r-project.org/) was used to generate all graphical presentations.

## Results

### 1. Morphological Changes in BGC-823 Cells Mimick Stromal Cells

Fibroblasts (FBs) and BGC-823 cells were cocultured at a ratio of 10∶1 for an additional 10 days after the cultured cells reached confluence. The cell mixture was then passaged by trypsinization with fresh fibroblasts (1×10^6^ cells/mL) (Figure 1.1). Dome formation in the BGC-823 cells was observed after the first passage. During the second and subsequent passages, suspended round-shaped cells appeared in the cultured system: some aggregated into clusters or clumps and were loosely connected to the surface of the fibroblasts. The parallel-cultured BGC-823 cells demonstrated only a few round-shaped cells at the base when the culture became overcrowded. The mixed suspended cells exhibited diverse morphological features and prolonged attachment after passage. These cells demonstrated either a cobblestone-like appearance similar to BGC-823 cells or short spindle-like features. Uniform, short, spindle-like cells were produced by the passage of the mixed suspended cells and dense normal fibroblasts. In contrast, passage of the supernatant of the BGC-823 cells only demonstrated debris after 24 hours of cultivation, in which small amounts of cells survived to form cobblestone-like clones similar to parental BGC-823 cells (Figure 1.3A–H).

**Figure 1:**
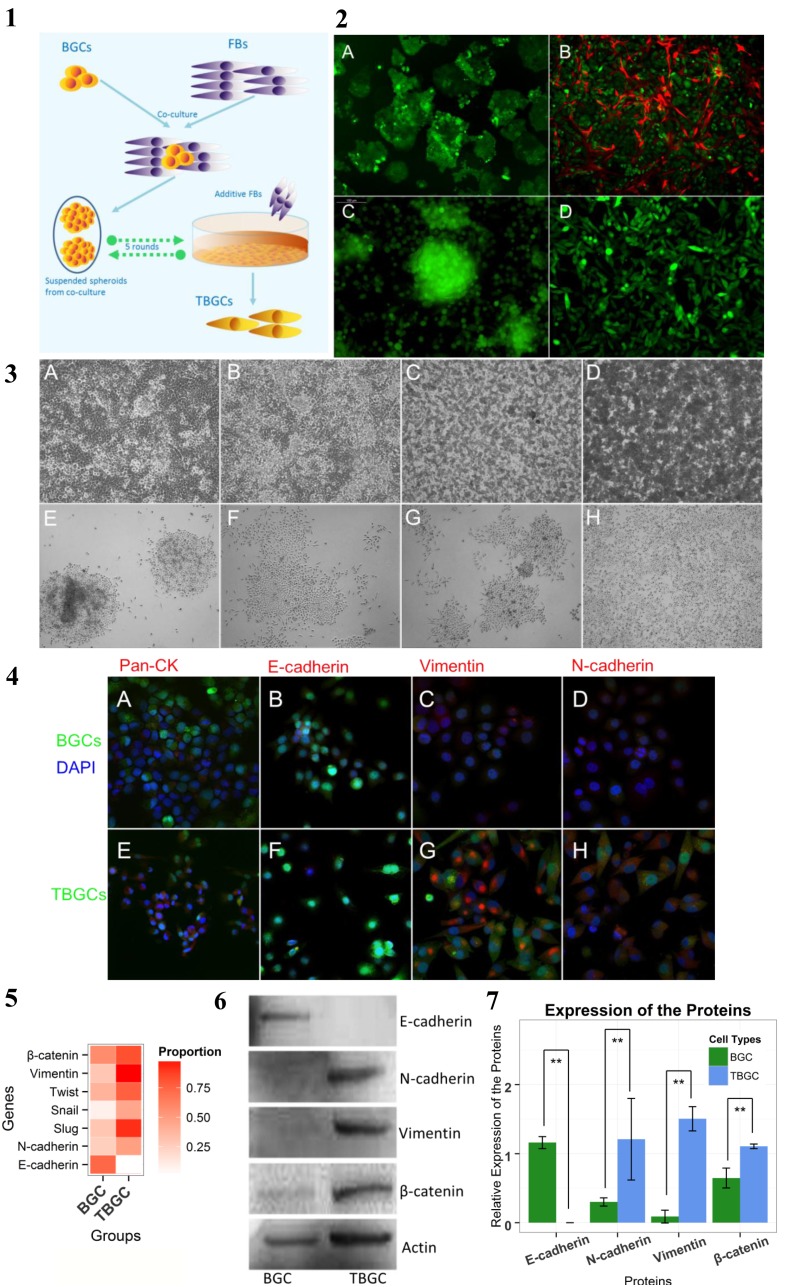
Morphological changes in BGC-823 cells were associated with the epithelial-mesenchymal-transition. **Figure 1.1.** Schematic of the experimental protocol. **Figure 1** **.2.** Origin of the supernatant cells. (A) GFP-labeled BGC-823 cells (green). (B) BGC-823 cells that were cocultured with fibroblasts (red). (C) BGC-823 cells grew into clumps and demonstrated round shapes in suspension. (D) TBGCs were induced by coculturing. Magnification 100×. **Figure 1** **.3.** Transformation from BGC-823 cells to TBGCs under a phase-contrast microscope. (A–D) BGC-823 cells in a dense culture system can form clusters and shed off cells into the suspension. (E–H) Passage of suspended tumor cells. **Figure 1** **.4.** Immunofluorescent staining of pan-CK (red), E-cadherin (red), vimentin (red) and N-cadherin (red). BGC-823 cells (above) and TBGCs (below) were labeled with GFP (green). The nucleus was stained with DAPI (blue). **Figure 1** **.5.** Heat plot of gene expression profiles analyzed using fluorescence quantitative RT-PCR. The depth of the color indicates relative gene expression. **Figure 1** **.6.** Western blots of proteins. **Figure 1** **.7.** Bar plot of protein expression determined using Western blot analysis. Bars denote the relative expression levels measured using IOD. Vertical lines denote standard differences. **p<0.01.

Subsequent experiments were conducted to determine the source of the suspended cells in the cocultured system. BGC-823 cells and fibroblasts were transfected with GFP-puro cassette and RFP-puro cassette, respectively, and named BGC-GFP and FB-RFP, respectively. After coculturing, BGC-GFP grew into multilayers and formed a dome-like structure. Meanwhile, the round-shaped or spheroid cells that were suspended in the media were transferred to culture plates and labeled with green fluorescence, demonstrating that long-term coculturing with fibroblasts induces BGC transformation. Round and spheroid cells that were suspended in the medium were also observed using green fluorescence and could be passaged without the addition of fibroblasts (Figure 1.2A–D). After several successive rounds of coculturing, transformed BGC-823 cells (TBGCs) were obtained that appeared more uniform, short, and spindle-like. These cells were then passaged without the addition of fibroblasts. Interestingly, these cells were more prone to form spheroid structures than the cells that grew to confluence and did not demonstrate morphological reversion to BGC-823 cells until >10–15 passages.

A previous study reported that fibroblasts inhibit the growth of other cells when cocultured *in vitro* by the mechanism of contact inhibition [4]. In our experiments, however, normal fibroblasts did not contact-inhibit the proliferation of BGC-823 cells. Instead, FB-RFPs gradually perished with BGC-GFP proliferation when culturing was extended to 10 days. Therefore, consecutive FB supplementation was required to passage and sustain the stable production of TBGCs. We also observed that when a small amount of TBGCs was added to the confluent fibroblast sheet, the tumor cells that grew outside were pushed away by the fibroblasts. In the center of the tumor colony, the cells were round-shaped, with just loose attachments to the base (Figure 1.3A–H).

### 2. Epithelial-mesenchymal Transition from BGC-823 Cell to TBGC

Real-time PCR was used to analyze mRNA expression. The results show that E-cadherin expression was remarkably downregulated along with the upregulation of vimentin in TBGCs. Meanwhile, the expressions of twist, snail, slug, and N-cadherin were all upregulated (Figure 1.5). Immunofluorescence staining and Western blot confirm E-cadherin loss and the increased expression of vimentin, N-cadherin, and β-catenin (Figure 1.4), thereby confirming that long-term epithelial-mesenchymal transition (EMT) occurs in TBGCs. E-cadherin is a proven hallmark of EMT and maintains cell-cell attachment in the epithelium. E-cadherin loss could lead to the tumor shedding more cells off from the tumor mass.

### 3. Proliferation, Invasion, and Mobility of TBGCs

TBGC proliferation was analyzed using the MTS assay. TBGCs demonstrated an accelerated proliferation rate (*p*<0.01) (Figure 2.1). Flow cytometry (see Materials and Methods S1) shows that 13% of TBGCs were retained in the S phase, in contrast to only 7% of BGC-823 cells (Figure S1.3). The scratching assay indicates that TBGC motility increased in comparison with paternal BGC-823 cells (Figure 2.2–2.3A–H). TBGC anastomosis required 3 days after the initiation of the scratches, whereas >4 days were needed for BGC-823 cells (*p*<0.05) (Figure 2.2).

**Figure 2:**
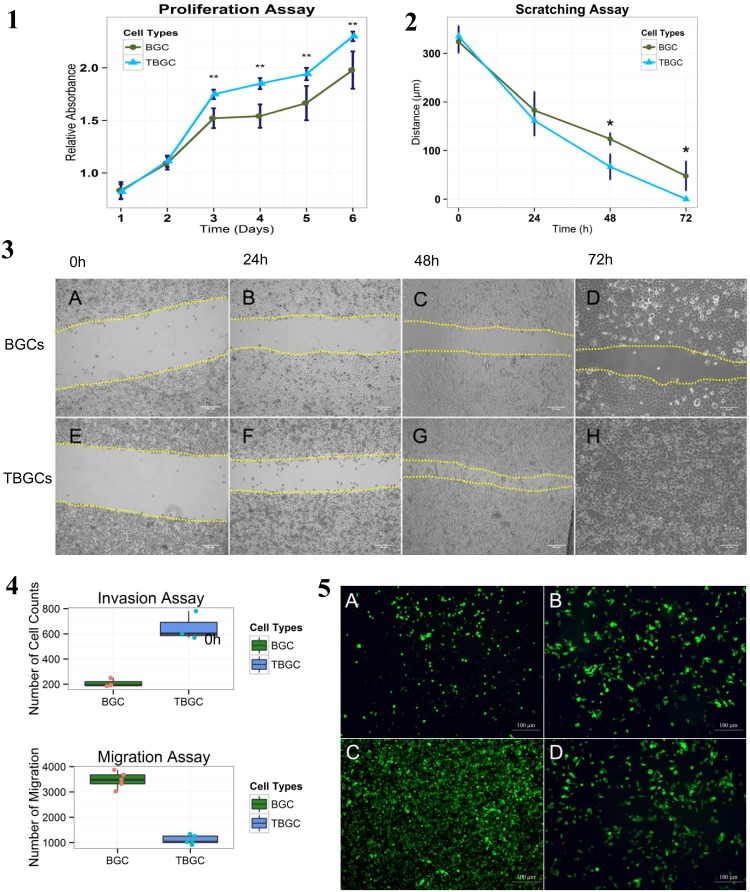
Proliferation, invasion, and mobility of TBGCs. **Figure 2.1.**Line plot of the proliferation assay. Vertical lines denote standard differences. **p<0.01. **Figure 2** **.2.** Line plot of the scratching assay. Vertical lines denote standard differences. **p<0.01. **Figure 2** **.3.** Scratching assay of BGC-823 cells (above) and TBGCs (below) under a phase-contrast microscope. (A–D, E–H) Time from initial to 72 hours. **Figure 2** **.4.** Box plot of the invasive and migration assays. Differences between BGC-823 cells and TGBCs are significant (p<0.01). **Figure 2** **.5.** The top 2 photos show the results of the invasive modified transwell assay with Matrigel. Representative images of the transwell invasion assays for (A) BGC and (B) TBGC. Cells invading the underside of the transwell insert are shown. The bottom 2 photos are representative images of the transwell migration assays for (C) BGC and (D) TBGC. Cells migrating to the underside of the transwell insert are shown.

The results of the Matrigel invasion assays show that TBGCs are more aggressive than BGC-823 cells. In total, 683.67±170.83 TBGCs infiltrated the Matrigel in comparison to 188.33±58.62 BGC-823 cells in the control group (*p*<0.01) (Figure 2.4, Figure 2.5A,B). However, TBGCs demonstrated a significant reduction in migrated cells: 2-fold less than BGC-823 cells according to the transwell migration assay (*p*<0.01) (Figure 2.4, Figure 2.5C,D). The suspended nature of the TBGCs might account for this reduction, the adherence rate was only 68.33±10.41% in TBGCs on Matrigel while 99.77%±6.25% in BGC-823 cells, demonstrated slow attachment to the Matrigel surface in TBGCs (Table S1).

To better mimic *in vivo* tumor behavior, collagen type I gel was used to ascertain the relationship between fibroblasts and the invasive capacities of BGC-823 cells and TBGCs. Collagen type I gel was prepared on the transwell inserts with fibroblasts (see Materials and Methods S1). Fibroblast-loaded gels were cultured for 7 days, and then GFP-labeled cancer cells were seeded onto the gel and cultured for the next 7 days. Inspection of the harvested gels revealed that the tumor cells had penetrated into the collagen gels. When the gels were loaded with fibroblasts, TBGCs exhibited more enhanced invasive capacity than BGC-823 cells, as indicated by the number of cells that infiltrated the gels (Figure S1.2).

### 4. TBGCs Exhibited Cisplatin Resistance

Next, we assessed the sensitivity of TBGCs to standard gastric cancer chemotherapeutics. Viable BGC-823 cells and TBGCs were determined after 24 hours of exposure to cisplatin (0, 20, 40, 60, 80 µM) by measuring the incorporation of MTS. The results demonstrate that TBGCs exhibited remarkable resistance to cisplatin, whereas few BGC-823 cells survived when the cisplatin concentration was elevated to 40 µM (*p*<0.001) (Figure 3.1). Viable TBGCs could even be detected when the cisplatin concentration was elevated up to 200 µM (data not shown). We performed cell-apoptosis analysis using flow cytometry to validate cisplatin resistance. The results show that the survival rate of TBGCs was about 42.40% compared with 13.80% for BGC-823 cells following treatment with 40 µM cisplatin for 24 hours (Figure 3.3–3.4). However, when treated with 5-FU, there were no significant differences in tumor inhibition between TBGCs and BGC-823 cells according to the MTS assay (Figure 3.2). Both tumor cells demonstrated chemoresistance to 5-FU (Figure 3.2).

**Figure 3:**
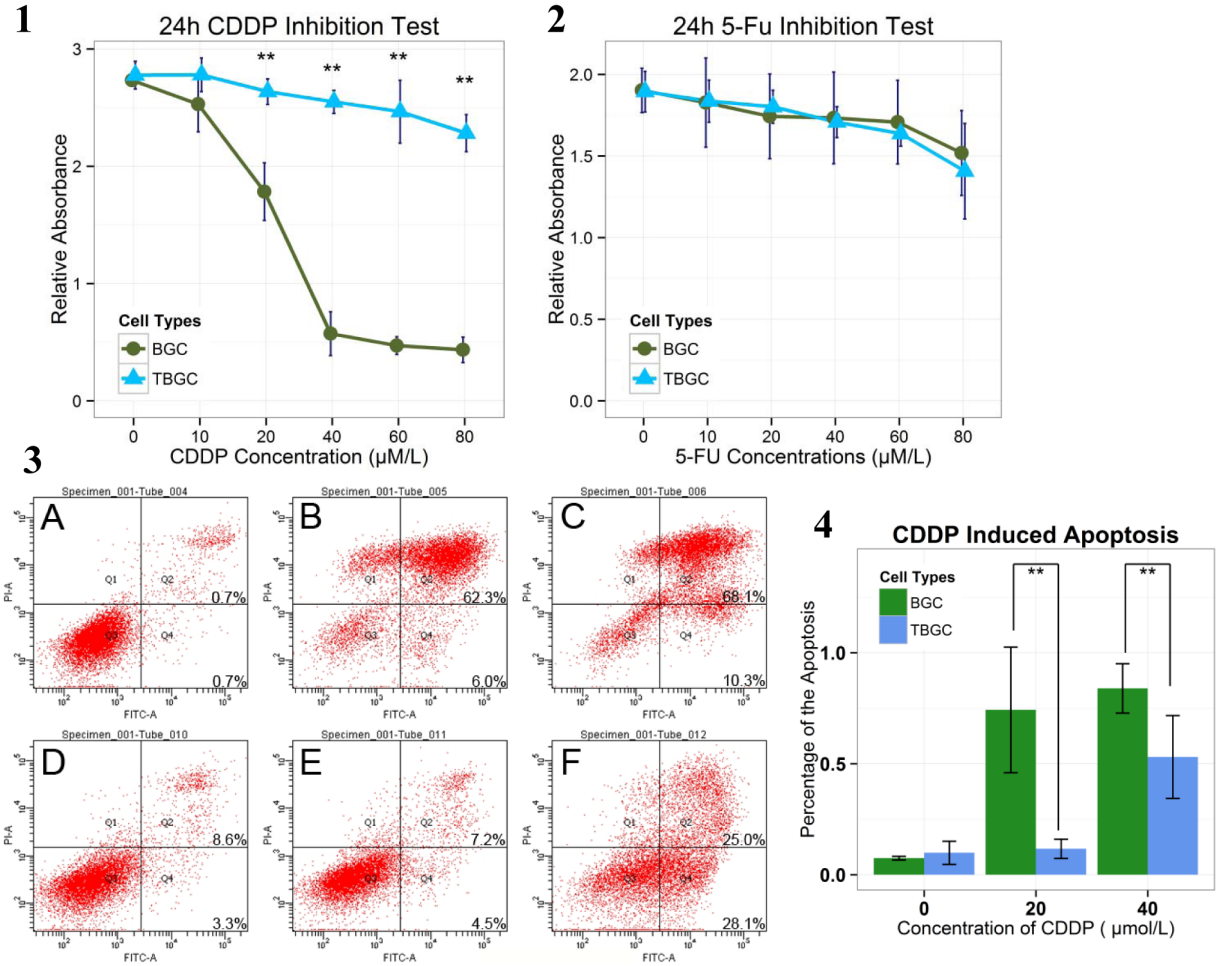
TBGCs exhibited cisplatin resistance. **Figure 3** **.1.** Twenty-four-hour line plot of the cisplatin-inhibition test. **p<0.01. **Figure 3** **.2.** Twenty-four-hour line plot of the 5-FU inhibition test. **Figure 3** **.3.** Flow cytometry was used to assess apoptosis in BGC and TBGC cells after exposure to different concentrations of cisplatin (20, 40 µM) for 24 hours. Cells were stained with Annexin V-FITC (marker of apoptosis) and propidium iodide (PI) (marker of dead cells). **Figure 3** **.4.** Bar plot of cisplatin-induced apoptosis in BGC-823 cells and TBGCs. Bar graph showing the percentage of apoptotic cells according to flow cytometry. **p<0.01 vs cells in the dimethyl sulfoxide (DMSO) control wells.

### 5. TBGCs Exhibit *In vivo* Lymph Node Propensity

To determine the *in vivo* distribution of TBGCs, 5×10^5^ BGC-823 cells or TBGCs were injected into the tail vein of BALB/c mice. Two weeks after injection, variance in auxiliary and cervical lymph nodes metastasis was observed in both groups, as indicated by assessment of a fluorescent tracer (Figure 4.3A–F). GFP-positive cells were found in the auxiliary and inguinal lymph node in both groups (Figure 4.3A, B, D, E). However, GFP-positive cells only appeared in the lungs of the mice in the BGC group (Figure 4.3C, F).

**Figure 4:**
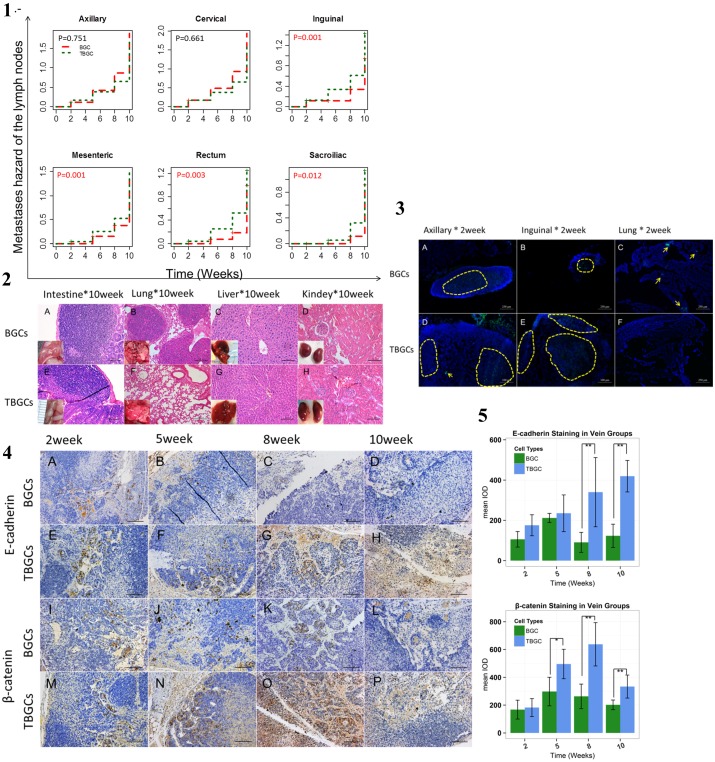
TBGCs exhibit in vivo lymph node propensity in the vein injection group. **Figure 4** **.1.** Cumulative risk of lymph nodes metastasis in different groups that received tail vein injections. **Figure 4** **.2.** HE staining (at 10 weeks) of organs in the group that received vein injections. Positivity was observed in the intestines of both groups and in the lungs of the BGC groups. No evident liver or kidney metastases were found. **Figure 4** **.3.** Fluorescent tracing of tumor cells at week 2 in the groups that received tail vein injections. Tumor cells were labeled with green fluorescence as indicated by the yellow arrowheads. The nucleus was stained with DAPI (blue). Magnification 100×. **Figure 4** **.4.** Immunohistochemical staining for E-cadherin and β-catenin in the lymph nodes of the groups that received tail vein injections at each time point. Magnification 200×. **Figure 4** **.5.** Bar plot of the relative expression levels of E-cadherin and β-catenin in the lymph nodes of the groups that received tail vein injections at each time point. **p<0.01; *p<0.05.

At week 5, in addition to extensive invasion into the lymph nodes, organ infiltration was also observed. More lymph node invasion was observed in the TBGC group than the BGC control group at each time point (Figure 4.1, Video S1). These lymph nodes were determined to be pan-CK positive (Figure S2.1). We also observed different TBGC and BGC distribution in the organs. TBGCs were limited to the gastroenteric tissues, whereas BGCs settled into the lungs and intestines (Figure 4.2A–H). At week 5, intestinal metastasis was first observed in 3/4 mice that were injected with TBGCs, whereas only 25% mice demonstrated visible intestinal metastases following BGC injection (Figure S2.3).

As the tumors developed, the mean number of intestinal TBGC metastases increased (5.6±3.911 *vs* 3.5±1.914 BGCs at week 8; 6.33±1.52 *vs* 5.6±1.342 at week 10) (Figure S4.2). Interestingly, 3 of 5 mice in TBGC group demonstrated megascopic neoplasms in the gastrum after 10 weeks, which was not observed in the BGC group. Megascopic lung metastases were found in 3 of 4 BGC mice at week 8. However, no visible TBGC lung metastases were observed at week 10 (Figure 4.2A–H). Microscopic inspection revealed the infiltration of TBGCs into the cervical and axillary lymph nodes as early as 1 week after tail vein injection, whereas BGCs required more time (3 weeks) to enter these lymph nodes (Figure 4.1). Five weeks after cell injection, lung infiltration was observed in the BGC group. However, no TBGCs were detected in organs until 10 weeks after injection (Figure 4.2B, F, Figure S2.3).

Immunohistochemical staining indicated the gradual increase of E-cadherin in the lymph nodes of the TBGC group, whereas E-cadherin expression slightly decreased in the BGC group (*p*<0.01) (Figure 4.4A–P). Differences were significant at 8 and 10 weeks, when E-cadherin expression was much higher in the TBGC group compared with the BGC group (Figure 4.5). The expression of β-catenin also increased with time in the TBGC group, which peaked at 8 weeks and then slightly decreased (Figure 4.5). The results also show increased β-catenin expression in the TBGC group at 5, 8, and 10 weeks (*p*<0.01) (Figure 4.5).

### 6. TBGCs Demonstrated Low Tumor Formation but High Lymph Node Metastasis Capacity in our Subcutaneous Tumorigenesis Model

Because tumor cells are more likely to form neoplasms when subcutaneously implanted, (GFP+)TBGCs and the BGCs were implanted by injecting 5×10^5^ viable tumor cells into the subcutaneous tissues on both sides of the chest. Nascent tumors in the BGC group were detected as early as 3 days post-implantation, and all mice in the BGC group developed tumors at the sites of implantation after 1 week of incubation (Figure 5.1A). It is striking that the implanted TBGCs yielded no undetectable tumors until 8 weeks (Figure 5.1, Video S2). Escalating the TBGC dose to 5–10×10^6^ implanted cells only led to the formation of small tumor clumps and the sluggish augmentation of tumor volume along with incubation time. In addition, mice in the BGC group became moribund, apparently because of increased tumor burden (data not shown).

**Figure 5:**
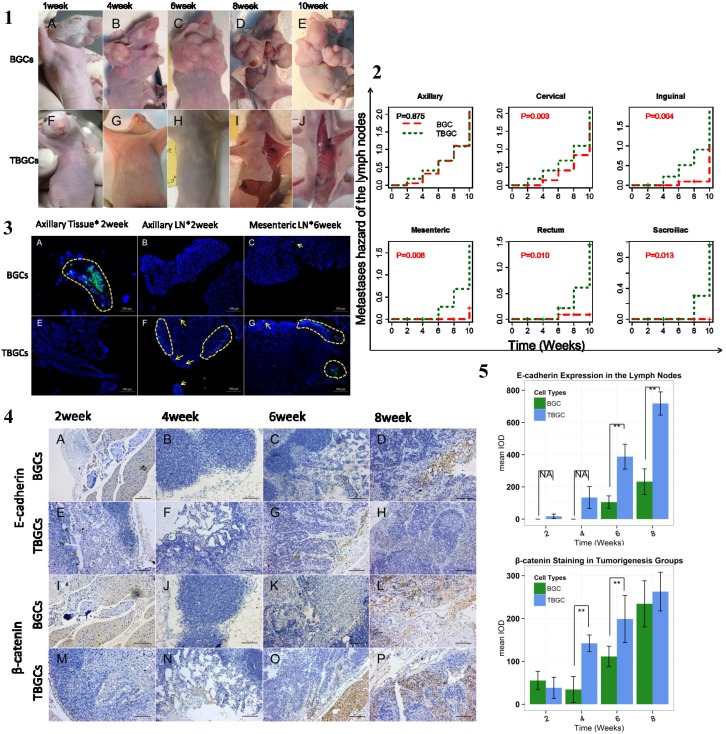
TBGCs demonstrated low tumor formation but high lymph node metastasis capacity in our subcutaneous tumorigenesis model. **Figure 5.1.**General tumorigenesis observations in the BGC and TBGC groups at each time point. **Figure 5** **.2.** Cumulative risks of lymph nodes metastasis in the different tumorigenesis groups. **Figure 5** **.3.** Fluorescent tracing of tumor cells at week 2 in the tumorigenesis groups. Tumor cells are labeled with green fluorescence and indicated by the yellow arrowheads. The nucleus was stained with DAPI (blue). **Figure 5** **.4.** Immunohistochemical staining for E-cadherin and β-catenin in the lymph nodes of the tumorigenesis groups at each time point. Magnification 200×. **Figure 5** **.5.** Bar plot of the relative expression of E-cadherin and β-catenin in the lymph nodes of the tumorigenesis groups at each time point. **p<0.01; *p<0.05. NA: no metastatic lymph nodes were observed in the BGC group.

Whole-body autopsies at each time interval show that TBGCs, instead of visible primary tumors, infiltrated the adjacent and distal lymph nodes, whereas BGC-generated tumor masses were confined to intact fibrotic capsules at the injection sites (Video S2). The regional global lymph nodes metastases in the TBGC group were found as early as 2 weeks after implantation, whereas regional lymph nodes metastases were detected at 4 weeks in the BGC group (Figure 5.2). Kaplan-Meier analysis shows that the TBGCs demonstrated greater risk of developing into lymph node metastasis than BGCs (Figure 5.2).

Extensive intestinal metastases were also evident in all mice in the TBGC group at 8 weeks and thereafter (Figure S4.1 Figure S4.4). The average number of intestinal nodules was 5.50±1.73 at week 8 and 7.33±1.79 at week 10. In contrast, only 1 mouse in the BGC group demonstrated 3 intestinal infiltrations at week 9 (Figure S4.1).

GFP tracing indicates local tumor formation in the BGC group at the injection site without regional lymph node infiltration. In contrast, TBGCs appeared in the axillary lymph nodes, but green fluorescence was not detected in tissues at the injection site (Figure 5.3). At week 6, TBGCs-GFP accumulated in the rectal and mesenteric lymph nodes. However, only 1 mouse with positive mesenteric lymph node metastasis was detected in the BGC group. pan-CK immunohistochemical staining indicates that TBGCs metastasized to regional (early) and distal lymph nodes (late), whereas BGCs only metastasized to regional lymph nodes (late) within the experiment period (Figure S4.5). TBGCs also metastasized to the intestines at a week when it was not found in the BGC group.

HE staining demonstrates that abnormal cells appeared in the regional lymph nodes during the early stages in the TBGC group and late stages in the BGC group (Figure S4.6). Immunohistochemical protein expression analysis at 2 weeks shows the downregulation of E-cadherin and β-catenin and enhanced vimentin expression in TGBC-LNs in comparison with BGC-neoplasia. These results were further confirmed by Western blot analysis of the tissue samples, which did not detect E-cadherin in TGBC-LNs; meanwhile, N-cadherin was also sharply downregulated in TGBC-LNs (Figure S3.3–3.4).

Immunohistochemical staining indicates the gradual increase of E-cadherin in the lymph nodes of the TBGC group as time progressed, whereas the expression of E-cadherin slightly decreased in the BGC group (*p*<0.01) (Figure 5.4). The differences were significant at weeks 2, 6, and 8 when E-cadherin expression was much higher in the TBGC group than the BGC group. β-catenin expression also increased with time in the TBGC group. The results also show high β-catenin expression in the TBGC group at weeks 4 and 6 (*p*<0.01) (Figure 5.4–5.5).

## Discussion

Previous studies have used indirect transwell culturing, conditional culture medium, and short-term coculturing and suggest that normal fibroblasts promote tumor growth and migration [5]. In this study, we explore the effects of normal fibroblasts on tumor cells using direct coculturing. Our results confirm that long-term EMT can induce tumor cells (TBGCs) from BGC-823 cells when placed in contact with dense normal fibroblasts. This phenomenon suggested that spontaneous EMT might occur when tumor cells come into contact with stromal cells at the front or after metastasizing to a new place.

Morphological changes were first observed in the culture dish when some of the BGC-823 cells grew into clumps or clusters that were suspended in the culture media. Passage of these suspended cells confirmed that these cells adhered poorly to the base, regardless of the components. However, when the cells settled down to the base, they demonstrated elongated mesenchymal features that grew individually. These morphological changes are associated with EMT [9]. The suspended cells become stable when cocultured with normal fibroblast for >5 rounds. Mesenchymal markers (vimentin and N-cadherin) were upregulated in TBGCs. EMT-related genes, such as snail, slug, and twist, were also upregulated. Moreover, the loss of E-cadherin expression, a hallmark of EMT [6], [7], was validated in TBGCs.

Previous studies report that TGFβ1 is the most common inducer of EMT in different cell systems [8]–[12]. In all cases, growth factors usually induced transient EMT after brief treatment by activating specific signaling pathways that finally activated some transcriptional EMT inducers [7]. Long-term EMT is often induced by the ectopic expression of select transcriptional EMT inducers or their regulators. Transcription factors act as direct transcriptional repressors of E-cadherin and other epithelial genes and mediate the entire EMT gene program [13]. However, these inducers might interfere with natural conditions and hamper the growth of tumor cells. Our experiment shows that coculturing with FBs might be an ideal alternative for inducing long-term EMT.

E-cadherin is the primary cell adhesion molecule in the epithelium. The switching of cadherin from loss of E-cadherin to gain of N-cadherin is a part of the EMT process [14]. E-cadherin loss has been observed in TBGCs, and the loss of this protein is associated with the promotion of invasion, metastasis, drug resistance [15], aggressive tumor phenotypes, and poor patient prognosis in many cancers [16]–[20]. Synergistic tumor suppressor activity in E-cadherin and p53 have been observed in a conditional mouse model of gastric cancer and associated with metastasis to the lymph nodes [21]. β-catenin is associated with EMT in bladder cancer [22] and poor prognosis in colorectal cancer patients [23]. Our observations indicate that β-catenin increases when BGC-823 cells are transformed to TBGCs in the both in the *in vitro* and in the animal model, in accordance with the previous studies of EMT.

Aside from conversion to CAFs, normal fibroblasts are inextricably associated with tumors at every stage of tumor progression [24]. Normal fibroblasts reportedly induce reversion of the malignant phenotype in human primary breast cancer [25], inhibit the growth of SGC-7901 gastric cancer cells [26], and inhibit proliferation and invasion in human breast cancer cells [27]. However, our observations indicate that fibroblasts generally undergo apoptosis when cocultured with BGC-823 cells in a single dish, which might have been caused by competitive inhibition within a limited survivable space. Some smaller, round-shaped tumor cells dissociated from BGC-823 and were suspended in the culture media. These cells subsequently became TBGCs. This phenomenon may be caused by the growth factors and the type I collagen secreted by the cocultured fibroblasts. Growth factors, such as TGF-β that are secreted from high-dense fibroblasts, might induce EMT via down-regulating E-cadherin and up-regulating vimentin expression [28] [29]. Also, some studies report that collagen type I contribute to enhanced tumor growth and invasive properties by disrupting E-cadherin-mediated cell adhesion [29].

Cisplatin, a platinum-based agent, is the standard first-line chemotherapy for patients with metastatic gastric cancer [30], [31]. The gene expression signature of acquired chemoresistance to cisplatin includes the upregulation of AKT1, EIF4B, RPS6 (mTOR pathway) (DNA repair and drug metabolism genes), and genes that are overexpressed in embryonic stem cell signatures [32]. Researchers have reported other related factors: glutathione-S-transferases [33], p27 [34], RhoGDI2 [35], miR-21 [36], ZEB1 [37], and SNAI1 [38]. Our observations confirm that EMT is involved in cisplatin chemoresistance, in accordance with EMT-related chemoresistance in other tumors [39], [40]. Furthermore, the induction of EMT by normal fibroblasts might be an intrinsic process that occurs in the body, especially when tumor cells penetrate through the basement membrane where they are more prone to undergo EMT and become drug resistant.

It is worth noting that TBGCs did not form tumors when subcutaneously injected, nor did they metastasize to the organs except the intestines and stomach when injected either intravenously or subcutaneously. In contrast to parental cell lines, TBGCs produced systemic lymph nodes metastases. We considered that, among other factors [41]–[43], EMT together with E-cadherin loss might lead to tumor cells that are unable to settle down *in situ* via adhesion. Subcutaneously injected cells were soon collected in the surrounding lymphatic ducts and were transported and partly retained in the nearby lymph nodes. Reversion of the tumor morphology might also account for the survival status of the mice in the TGBC groups, especially the tumorigenesis group. However, it is also likely that the tumor cells were dormant, could develop tumorigenesis, and become virulent when they settle down and regain E-cadherin expression.

## Conclusion

Our experiment induced TBGCs from BGC-823 cells by coculturing with normal human fibroblasts. The process of TBGC generation was very similar to the *in vivo* tumors that were in contact with stromal cells. The loss of tumorigenesis and increased lymph node propensity indicate that TBGCs are mainly involved in metastasis and merit further study.

## Supporting Information

Figure S1 **Fibroblasts tracing in the co-culture system and the invasion and cell cycle of the TBGCs. Figure S1.1** Fibroblasts tracing in the co-culture system. The fibroblasts diminished when co-cultured with BGC-823 cells indicated by the red fluorescence reduced with the passages (A,B,C,D). A: initial; B: co-cultured 1 week; C: passage 1; D: passage 2. This co-culture system was passaged without fibroblasts add-in. Magnification×100 **Figure S1.2** Type I collagen based three dimensional culture of BGC-823 cells and TBGCs. A: HE staining of the BGC-823 cells cultured in the FB-loaded collagen gel for 1 week; B: Immunofluorescence staining for pan-CK(red) for BGC-823 cells; C: HE staining of the TBGCs cultured in the FB-loaded collagen gel for 1 week; D: Immunofluorescence staining for pan-CK(red) for TBGCs. Tumor cells were labeled with green fluorescence and indicated withyellow arrowheads. Nuclear was stained with DAPI (blue). **Figure S1.3** Cell cycle experiment by flow cytometry. BGC. FB denotes the cells are harvested from the co-culture BGC-823 cells and fibroblasts which has grew to 10-day-confluence; BGC. sup denotes the cells are harvested from culture suspension of BGC-823 cells; Sup denotes the cells are harvested from co-culture suspension of BGC-823 cells and fibroblasts.
(TIF)

Figure S2 **TBGCs exhibit in vivo lymph node propensity and intestinal metastasis in the vein injection group. Figure S2.1** Immunohistochemistry staining for pan-CK in the lymph nodes in the vein injection group at each time point. Magnification×100. **Figure S2.2** HE staining for the lymph nodes metastases in the vein injection group at each time point. Magnification×200 **Figure S2.3** Cumulative risk of the intestinal and lung metastases in different vein injection groups.
(TIF)

Figure S3 **Epithelial-mesenchymal transition in the subcutaneous tumor group at 2 week. Figure S3.1.** Immunohistochemistry study proteins expression in situ in the tumorigenesis groups at 2 week time point. In the BGC group, the specimen were harvested from the tumor mass. In the TBGC group, the specimen were harvested from the enlarged lymph node in the vicinity. **Figure S3.2.** Bar-plot of the Figure S3.1. **p<0.01. **Figure S3.3.** Western blot of the proteins expression in situ in the tumorigenesis groups at 2 week time point. In the BGC group, the specimen were harvested from the tumor mass. In the TBGC group, the specimen were harvested from the enlarged lymph node in the vicinity. **Figure S3.4.** Bar-plot of the Figure S3.3. **p<0.01.
(TIF)

Figure S4 **TBGCs demonstrated high lymph nodes and intestinal metastasis in the subcutaneous tumorigenesis model. Figure S4.1.** Bar-chart of the number of intestinal metastasis of BGCs and TBGCs in the tumorigenesis groups at each time point. The vertical lines indicate 95% confidence interval. **Figure S4.2.** Bar-chart of the number of intestinal metastasis of BGCs and TBGCs in the tail-vein injection groups at each time point. The vertical lines indicate 95% confidence interval. **Figure S4.3.** The representative macroscopic appearance of intestines metastases of mice bearing TBGCs at 6 week in the tumorigenesis group (A), while none was detected in the BGCs control until the end of the experiment (10 week). The sections of intestine metastases were stained with HE. Magnification×200. **Figure S4.4.** Cumulative hazard of intestinal metastasis in the tumorigenesis groups of BGC and TBGC. **Figure S4.5.** Immunohistochemistry staining for pan-CK in the lymph nodes in the tumorigenesis groups at early and later stage. Magnification×100. **Figure S4.6.** HE staining for the lymph nodes metastases in the tumorigenesis group at each time point. Magnification×200.
(TIF)

Table S1 **The adherence rate of the tumor cells on Matrigel.**
(DOCX)

Materials and Methods S1 **Supplementary Materials and Methods.**
(DOCX)

Video S1 **TBGC vein-injection 10 w.** Macroscopic inspection of the metastases in vein injection of TBGC group at 10 week time point.
(MOV)

Video S2 **TBGC tumorigenesis 6 w.** Macroscopic inspection of the metastases in subcutaneous tumorigenesis of TBGC group at 6 week time point.
(MOV)
