# Supplementary figures and images for: Normal Fibroblasts Induce E-Cadherin Loss and Increase Lymph Node Metastasis in Gastric Cancer
Source: PLoS One. 2014 May 20;9(5):e97306. doi: 10.1371/journal.pone.0097306 (PMC4028202; doi:10.1371/journal.pone.0097306)

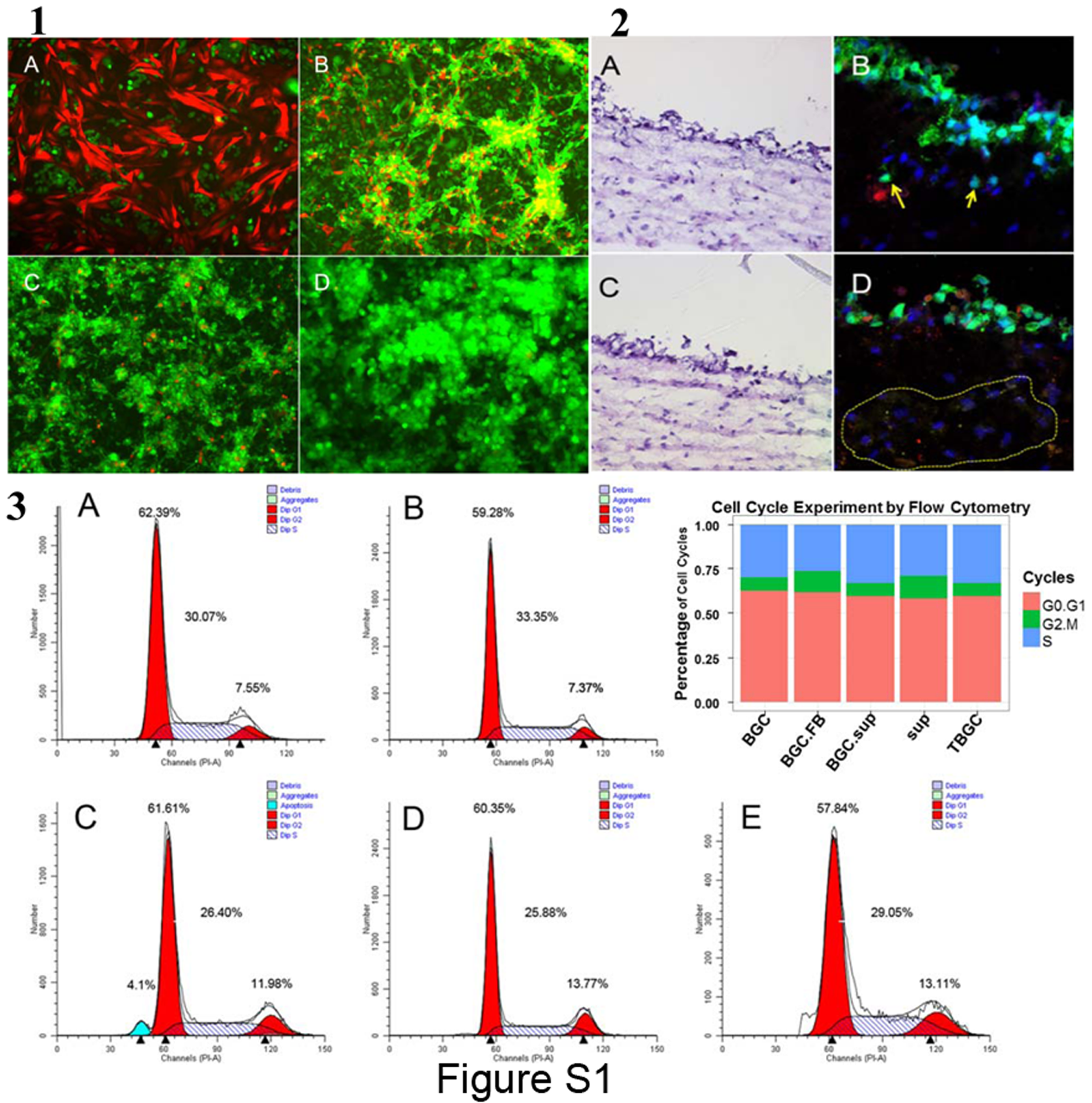

Supplement: Figure S1 — Fibroblasts tracing in the co-culture system and the invasion and cell cycle of the TBGCs. Figure S1.1 Fibroblasts tracing in the co-culture system. The fibroblasts diminished when co-cultured with BGC-823 cells indicated by the red fluorescence reduced with the passages (A,B,C,D). A: initial; B: co-cultured 1 week; C: passage 1; D: passage 2. This co-culture system was passaged without fibroblasts add-in. Magnification×100 Figure S1.2 Type I collagen based three dimensional culture of BGC-823 cells and TBGCs. A: HE staining of the BGC-823 cells cultured in the FB-loaded collagen gel for 1 week; B: Immunofluorescence staining for pan-CK(red) for BGC-823 cells; C: HE staining of the TBGCs cultured in the FB-loaded collagen gel for 1 week; D: Immunofluorescence staining for pan-CK(red) for TBGCs. Tumor cells were labeled with green fluorescence and indicated withyellow arrowheads. Nuclear was stained with DAPI (blue). Figure S1.3 Cell cycle experiment by flow cytometry. BGC. FB denotes the cells are harvested from the co-culture BGC-823 cells and fibroblasts which has grew to 10-day-confluence; BGC. sup denotes the cells are harvested from culture suspension of BGC-823 cells; Sup denotes the cells are harvested from co-culture suspension of BGC-823 cells and fibroblasts. (TIF) [file pone.0097306.s001.tif]

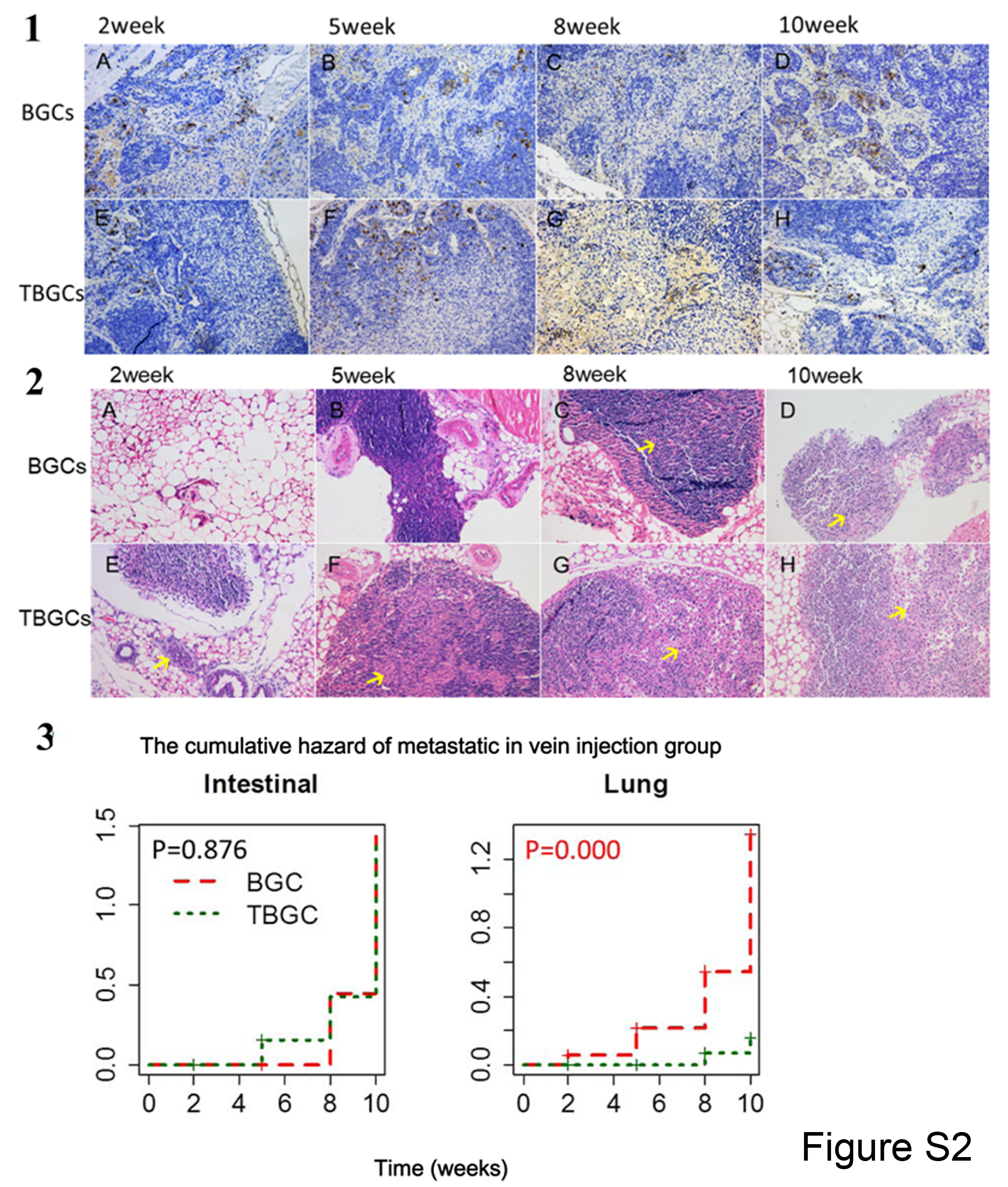

Supplement: Figure S2 — TBGCs exhibit in vivo lymph node propensity and intestinal metastasis in the vein injection group. Figure S2.1 Immunohistochemistry staining for pan-CK in the lymph nodes in the vein injection group at each time point. Magnification×100. Figure S2.2 HE staining for the lymph nodes metastases in the vein injection group at each time point. Magnification×200 Figure S2.3 Cumulative risk of the intestinal and lung metastases in different vein injection groups. (TIF) [file pone.0097306.s002.tif]

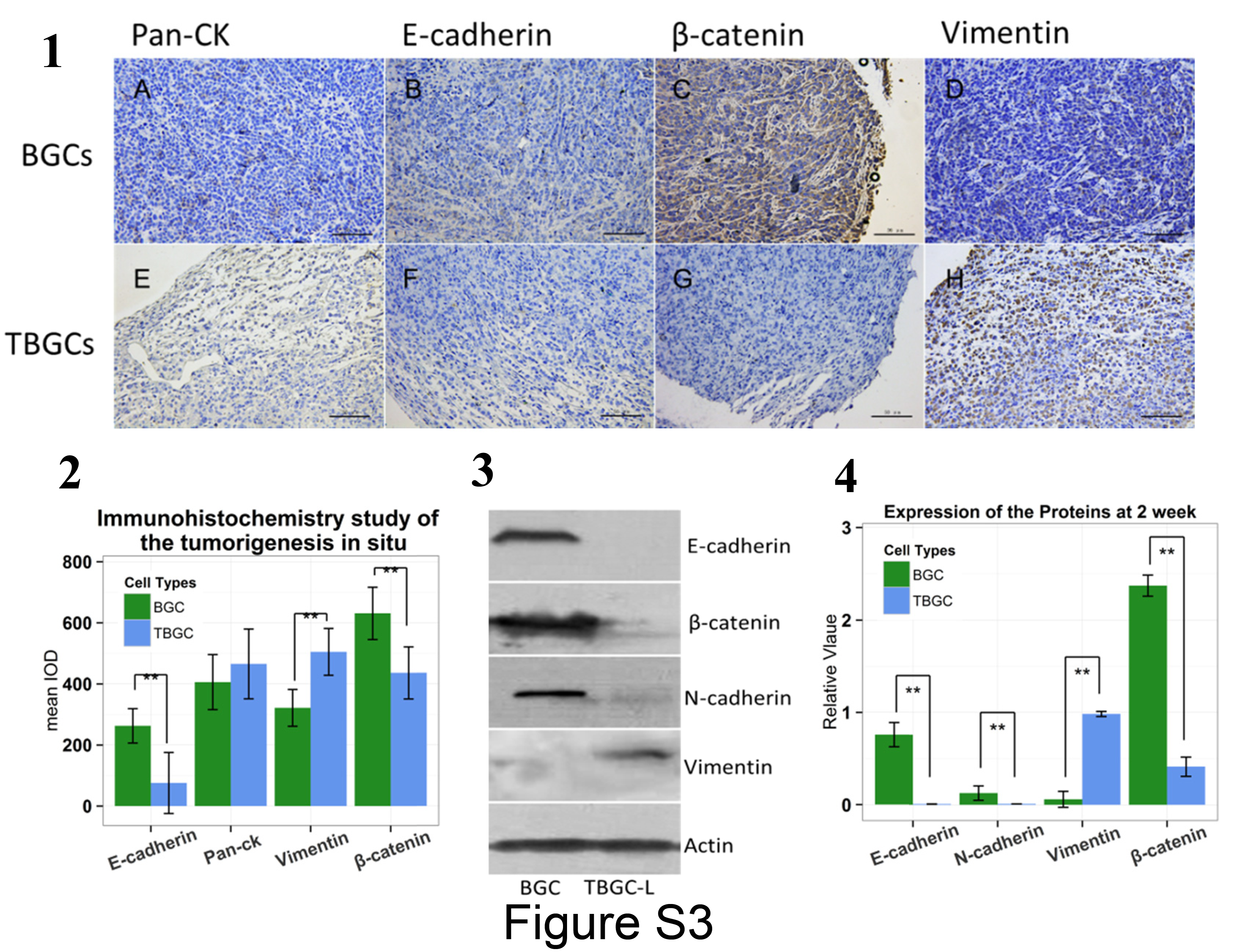

Supplement: Figure S3 — Epithelial-mesenchymal transition in the subcutaneous tumor group at 2 week. Figure S3.1. Immunohistochemistry study proteins expression in situ in the tumorigenesis groups at 2 week time point. In the BGC group, the specimen were harvested from the tumor mass. In the TBGC group, the specimen were harvested from the enlarged lymph node in the vicinity. Figure S3.2. Bar-plot of the Figure S3.1. **p<0.01. Figure S3.3. Western blot of the proteins expression in situ in the tumorigenesis groups at 2 week time point. In the BGC group, the specimen were harvested from the tumor mass. In the TBGC group, the specimen were harvested from the enlarged lymph node in the vicinity. Figure S3.4. Bar-plot of the Figure S3.3. **p<0.01. (TIF) [file pone.0097306.s003.tif]

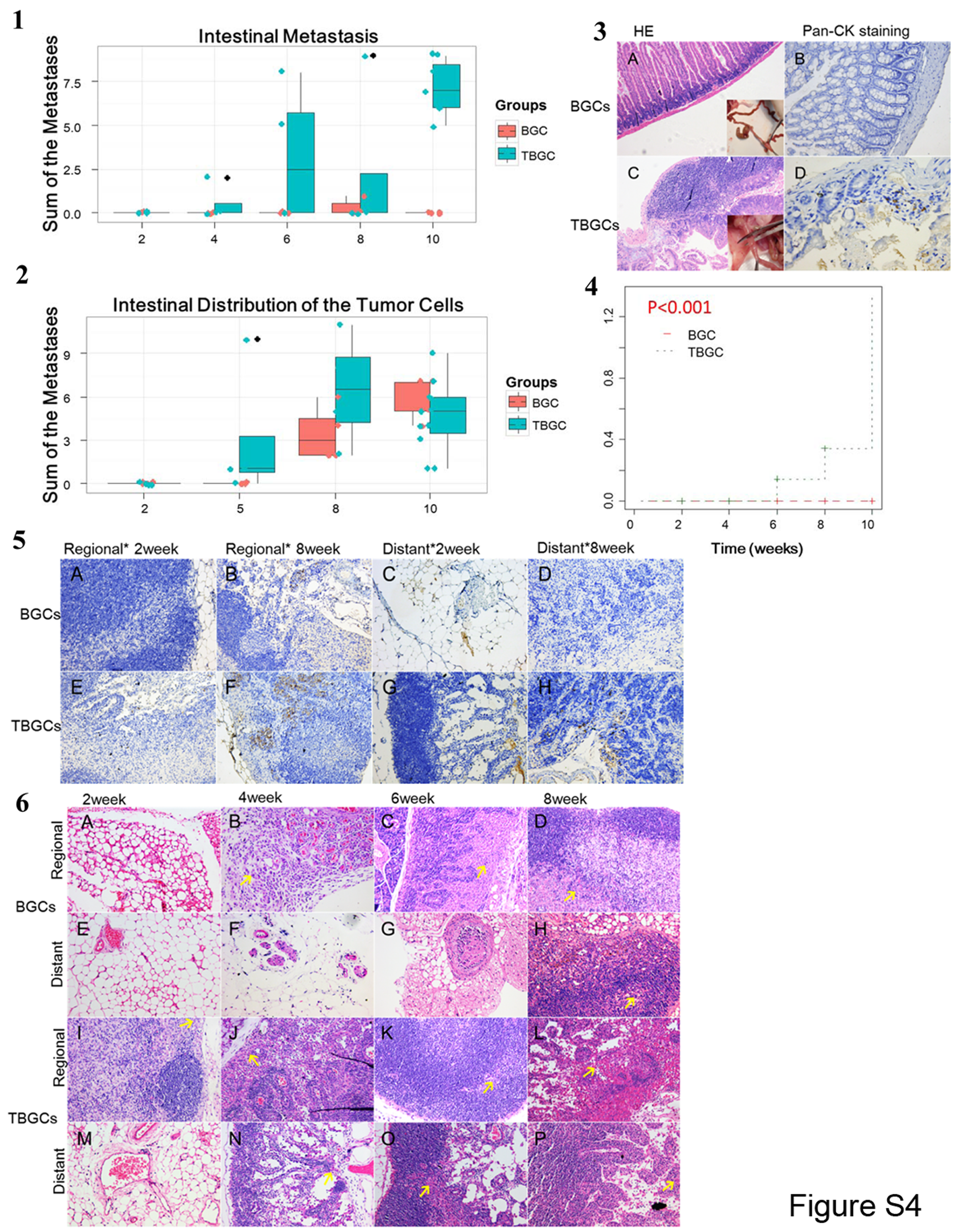

Supplement: Figure S4 — TBGCs demonstrated high lymph nodes and intestinal metastasis in the subcutaneous tumorigenesis model. Figure S4.1. Bar-chart of the number of intestinal metastasis of BGCs and TBGCs in the tumorigenesis groups at each time point. The vertical lines indicate 95% confidence interval. Figure S4.2. Bar-chart of the number of intestinal metastasis of BGCs and TBGCs in the tail-vein injection groups at each time point. The vertical lines indicate 95% confidence interval. Figure S4.3. The representative macroscopic appearance of intestines metastases of mice bearing TBGCs at 6 week in the tumorigenesis group (A), while none was detected in the BGCs control until the end of the experiment (10 week). The sections of intestine metastases were stained with HE. Magnification×200. Figure S4.4. Cumulative hazard of intestinal metastasis in the tumorigenesis groups of BGC and TBGC. Figure S4.5. Immunohistochemistry staining for pan-CK in the lymph nodes in the tumorigenesis groups at early and later stage. Magnification×100. Figure S4.6. HE staining for the lymph nodes metastases in the tumorigenesis group at each time point. Magnification×200. (TIF) [file pone.0097306.s004.tif]
