# Supplementary material for: Normal Fibroblasts Induce E-Cadherin Loss and Increase Lymph Node Metastasis in Gastric Cancer
Source: PLoS One. 2014 May 20;9(5):e97306. doi: 10.1371/journal.pone.0097306 (PMC4028202; doi:10.1371/journal.pone.0097306)
Supplement: Table S1 — The adherence rate of the tumor cells on Matrigel. (DOCX) [file pone.0097306.s005.docx]

Table S1.The adherence rate of the tumor cells on Matrigel

| Items | Total cells | Matrigel (mean) | Adherence Rate(%) |
| --- | --- | --- | --- |
| BGCs | 100000 | 99776.25 | 99.77±6.25 |
| TBGCs | 100000 | 68330.41 | 68.33±10.41 |

Notes: the *p* value was less than 0.0001.
