# Supplementary material for: Normal Fibroblasts Induce E-Cadherin Loss and Increase Lymph Node Metastasis in Gastric Cancer
Source: PLoS One. 2014 May 20;9(5):e97306. doi: 10.1371/journal.pone.0097306 (PMC4028202; doi:10.1371/journal.pone.0097306)
Supplement: Materials and Methods S1 — Supplementary Materials and Methods. (DOCX) [file pone.0097306.s006.docx]

**Supplementary**

**Transfection experiment**

Human gastric cancer cell line BGC-823 and the normal dermal fibroblasts at exponentially growing stage were seeded in the 6-well plate. When grown to 70%-80% confluence, the culture medium of BGC-823 and fibroblasts was replaced with 2 ml DMED high glucose containing 10μl plenti-GFP /plenti-RFP lentivirus respectively (GenePharma, China), and supplemented with 6 μl polybrene (GenePharma, China). At 24-hr post-transfection, fresh culture medium took place for further culturing and passaging. Transfected cells were then selected by culture medium containing 1 μg/ml puromycin for 24h which were confirmed under fluorescence microscope.

**Cell cycle assay**

Exponentially growing cells (1×10^5^) were harvested and fixed by -20 ˚C precooling 70% ethanol in -20˚C overnight then transferred to cell cycle kit (Keygen Biotech, China). Those cells were re-suspended in 500 µl binding buffer and added 100 µg/ml RNase for 30 min in 37˚C, then 5µl PI was added and the cell suspension were incubated in 37˚C, 30 min, away from light. Analysis was carried out using a flow cytometry (FACSCalibur™, Becton Dickinson, USA). The percentage of each phase was present in histograms.

**The organotypical culture**

For the collagen gel preparation, all steps were performed on ice. 1 ml Collagen solutions were prepared containing precooled 0.1 ml of 10×PBS, 0.69 ml of serum free DMEM with or witho E-cad ut tumor cells and 0.2 ml of rat tail collagen I (5mg/ml), which was neutralized with 23 µL of 1 mol/L NaOH. 300 µl Collagen type I (1mg/ml) mixed with or without fibroblasts (1×10^6^ /well) carefully were transferred into 12-well Transwell inserts(8-μm pore size polycarbonate membrane, Corning) which were gently polymerized at 37°C/5% CO2, 30 minutes. After 7-day cultivation, tumor cells (1×104 /well) were plated above the fibroblast-collagen or collagen layer. The culture medium was replaced every other day and maintained up to another 7 days. 3D gel on the chamber slides were fixed with 4% formaldehyde overnight at 4°before frozen section.

**Primer**

E-cadherin-F: TGCCCAGAAAATGAAAAAGG

E-cadherin-R: GTGTATGTGGCAATGCGTTC

N-cadherin-F: ACAGTGGCCACCTACAAAGG

N-cadherin-R: CCGAGATGGGGTTGATAATG

Vimentin-F: GAGAACTTTGCCGTTGAAGC

Vimentin-R: GCTTCCTGTAGGTGGCAATC

β-catenin-F: GCGTGGACAATGGCTACTCAAG

β-catenin-R: TATTAACTACCACCTGGTCCTC

GAPDH-F: ACCCAGAAGACTGTGGATGG

GAPDH-R: TCTAGACGGCAGGTCAGGTC
